# RIsearch: fast RNA–RNA interaction search using a simplified nearest-neighbor energy model

**DOI:** 10.1093/bioinformatics/bts519

**Published:** 2012-08-24

**Authors:** Anne Wenzel, Erdinç Akbaşli, Jan Gorodkin

**Affiliations:** ^1^Center for non-coding RNA in Technology and Health, ^2^Department of Veterinary Clinical and Animal Sciences, University of Copenhagen, Grønnegårdsvej 3, DK-1870 Frederiksberg, Denmark and ^3^Software Development Group, University of Copenhagen, Rued Langgaards Vej 7, DK-2300 Copenhagen S, Denmark

## Abstract

**Motivation:** Regulatory, non-coding RNAs often function by forming a duplex with other RNAs. It is therefore of interest to predict putative RNA–RNA duplexes *in silico* on a genome-wide scale. Current computational methods for predicting these interactions range from fast complementary-based searches to those that take intramolecular binding into account. Together these methods constitute a trade-off between speed and accuracy, while leaving room for improvement within the context of genome-wide screens. A fast pre-filtering of putative duplexes would therefore be desirable.

**Results:** We present RIsearch, an implementation of a simplified Turner energy model for fast computation of hybridization, which significantly reduces runtime while maintaining accuracy. Its time complexity for sequences of lengths *m* and *n* is 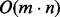 with a much smaller pre-factor than other tools. We show that this energy model is an accurate approximation of the full energy model for near-complementary RNA–RNA duplexes. RIsearch uses a Smith–Waterman-like algorithm using a dinucleotide scoring matrix which approximates the Turner nearest-neighbor energies. We show in benchmarks that we achieve a speed improvement of at least 2.4× compared with RNAplex, the currently fastest method for searching near-complementary regions. RIsearch shows a prediction accuracy similar to RNAplex on two datasets of known bacterial short RNA (sRNA)–messenger RNA (mRNA) and eukaryotic microRNA (miRNA)–mRNA interactions. Using RIsearch as a pre-filter in genome-wide screens reduces the number of binding site candidates reported by miRNA target prediction programs, such as TargetScanS and miRanda, by up to 70%. Likewise, substantial filtering was performed on bacterial RNA–RNA interaction data.

**Availability:** The source code for RIsearch is available at: http://rth.dk/resources/risearch.

**Contact:** gorodkin@rth.dk

**Supplementary information:** Supplementary data are available at *Bioinformatics* online.

## 1 INTRODUCTION

Non-coding RNA (ncRNA) form an abundant class of genes involved in both regulation and housekeeping functions, often in complexes with proteins and/or through interactions with other RNAs (Amaral *et al.*, 2008). The potential for ncRNAs is becoming apparent, e.g. in the mammalian genome where the protein coding regions account for ∼1.2% (International Human Genome Sequencing Consortium, 2004) while the majority of the genome is transcribed (The ENCODE Project Consortium, 2007). Even in smaller genomes, such as fungi strains of *Aspergillus*, ‘only’ 45–50% of the genome encodes proteins (Galagan *et al.*, 2005), leaving plenty of room for ncRNAs which could hold the potential for improvements to microbial industrial production, such as has already been investigated in *Streptomyces* (D’Alia *et al.*, 2010). Also in mammalian production systems such as Chinese hamster ovary cell lines, ncRNAs receive increasing attention (Barron *et al.*, 2011).

Computational screens for structured RNAs result in thousands of candidates on a genome-wide scale and it is of interest to predict possible RNA interaction partners of these sequences (Gorodkin *et al.*, 2010; Gorodkin and Hofacker, 2011). These candidates are predicted from sequence and structure-based alignments, by using a combination of thermodynamic and evolutionary constraints (such as compensating base pair changes) (Pedersen *et al.*, 2006; Torarinsson *et al.*, 2006, 2008; Washietl *et al.*, 2005; Weinberg *et al.*, 2007). A principal problem, however, is to obtain experimental data for each of these candidates, such as the full-length RNA sequence and its function. Another factor is that many ncRNAs are expressed at extremely low levels. For example, the regulatory antisense RNA of the GAL10 operon in yeast is functional and expressed as one copy per 14 cells (Houseley *et al.*, 2008). Coping with such low expressed ncRNAs on a high-throughput experimental scale is still an intractable task.

One approach toward functional analysis of ncRNA candidates is to search for possible interactions with other RNAs, as a substantial class of ncRNAs function by duplex formation with other RNAs, of which microRNAs (miRNAs) are a popular example. However, not only small ncRNAs interact by base pairing, but also long ncRNAs. For example, Staufen 1-mediated messenger RNA decay (SMD) can be initiated by imperfect base pairing between Alu elements in a lncRNA and in the 3′-UTR of an SMD target (Gong and Maquat, 2011). Translational regulation by short RNAs (sRNAs) is also a common mechanism existing in bacteria (Waters and Storz, 2009). A well-known example is the MicC-ompC messenger RNA (mRNA) interaction causing translational repression (Chen *et al.*, 2004; Vogel and Papenfort, 2006).

It is furthermore of interest to use the homology information from multiple structural alignments of ncRNA candidates for interaction prediction. Recent computational approaches that address this are PETcofold (Seemann *et al.*, 2011) and ripalign (Li *et al.*, 2011). Other methods that work on pairs of single sequences include PairFold (Andronescu *et al.*, 2005), RNAup (Mückstein *et al.*, 2006) and inteRNA (Alkan *et al.*, 2006). However, such approaches which take both *intra*- and *inter*molecular base pairings into account seem in general to be less suitable for genome-wide interaction screens.

It is therefore relevant to develop fast methods to search for putative RNA–RNA interactions not only for existing ncRNA candidates, but also to find novel RNAs which, for example, hold the potential to be lineage specific or perform lineage-specific functions (Bentwich *et al.*, 2005; Ohhata *et al.*, 2011).

The computational prediction of RNA–RNA interactions is a rapidly growing research area. As discussed in Seemann *et al.* (2011) , several classes of algorithms with increasing complexity in time and memory were developed. Here, we consider algorithms that use a thermodynamic energy model for predicting intermolecular interactions, while ignoring intramolecular structures. An early method was RNAhybrid (Rehmsmeier *et al.*, 2004) which makes use of the full energy model, only excluding intramolecular base pairings and multiloops. This yields a complexity of 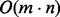 in memory and 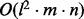 in time, with *m* and *n* being the lengths of query and target, respectively, and *l* the maximum loop length. Hodas and Aalberts (2004) explicitly noted the analogy between Smith–Waterman sequence alignment (Smith and Waterman, 1981) and intermolecular RNA pairing. Their implementation in BINDIGO handles different loop types with different states and dynamic programming (DP) matrices. A recent and faster method is RNAplex (Tafer and Hofacker, 2008; Tafer *et al.*, 2011), which uses a simpler energy model in the first ‘scanning phase’. It approximates larger loops with a linear model, thereby discarding the length-dependent term and thus the factor 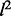 in time complexity. It also reduces memory consumption to a linear scaling. In a second step, the full energy model can be used on sub-sequences to refine potential binding sites. Even though RNAplex decreases time complexity while maintaining a thermodynamic model, applying it to genome-wide screens is still a computationally demanding task.

Here, we simplify the energy model even further, with the goal of designing a screening method for RNA–RNA interactions that can be used as a pre-filter for computationally complex methods in genome-wide screens. Consequently, our goal here is to search for near-complementary regions. To our knowledge, the fastest tool for such a task is RNAplex which recently was extended to use the information from multiple sequence alignments as well as pre-computed accessibility profiles. However, when searching for near-complementary regions, the energy model of RNAplex can be simplified further. Here, we introduce RIsearch, which implements a simpler state model that essentially takes all stacked base pairs into account, but averages costs over loop openings, internal loops and bulges. This is realized by an alignment-like algorithm that treats dinucleotides as the elementary unit of a 36 × 36 scoring matrix. An additional layer of heuristics can be used for genome-wide searches, which reduces the search space by first identifying short stretches of complementarity and extending those with RIsearch. It is beyond the scope of this work to fully address this additional step. One major consideration here is the G–U wobble, which has been addressed earlier, e.g. in GUUGle (Gerlach and Giegerich, 2006).

## 2 MATERIALS AND METHODS

### 2.1 The algorithm

The underlying algorithm of RIsearch can be seen as an extension of the Smith–Waterman–Gotoh algorithm (Gotoh, 1982; Smith and Waterman, 1981) for local sequence alignment. To find putative interaction sites, we look for complementarity rather than similarity/identity. A similar idea has been used in the so-called ‘individual base pair model’ introduced by TargetRNA (Tjaden *et al.*, 2006; Tjaden, 2008). The crucial difference is that our scoring scheme is based on dinucleotides instead of single nucleotides. This allows us to reflect the main properties of the nearest-neighbor free energy model, which is widely applied for RNA folding (see below for details on the scoring scheme). It can also be considered as a scoring scheme taking di-residue substitutions into account with gaps being an explicit part of the scoring scheme (Akbasli, 2008). An alignment approach making use of di-residue substitutions, but with other gap scoring was introduced as well (Crooks *et al.*, 2005).

**Fig. 1.**
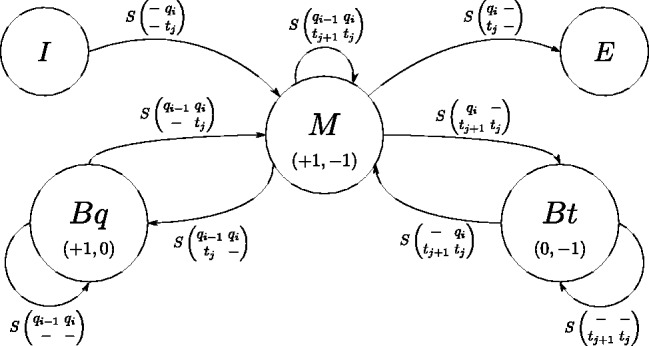
The flow in the three-state model. This state model has been (developed for and) proven useful before in the pairwise alignment of amino acid sequences using doublets, hereby taking into account correlation of neighboring residues (Akbasli, 2008). Here, the dashes indicate bulges or asymmetric internal loops, but are equivalent to gaps when the state model is applied to sequence alignments. States are represented by circles, transitions by connecting arcs. The number of pairs in the circles indicate the index increments to reach that state, e.g. for the *Bq*-state (bulge in query) only the q(uery) index is incremented, thus ‘(+1,0)’, while the *M*-state is reached by (mis)matching two residues, so both indices are updated. In a DP matrix, this corresponds to moving diagonally for transitioning into the *M*-state, and horizontally/vertically for the *B*-states. Indices along the t(arget) sequence are decremented as the two interacting RNA strands run in opposite directions. See recursion in the text for further description

When neglecting intramolecular base pairing, the only structural elements that need to be considered are stacked pairs, bulges and interior loops. Contributions from dangling ends and terminal mismatches are excluded to keep the algorithm simple and fast. We use a three-state model (Fig. 1), with an *M*-state for consecutive matches (stacked pairs) and mismatches (interior loops) and *Bq*/*Bt* states for gaps (bulges) in either sequence (query *q* or target *t*). All interactions come from the I(nitiation)-state and terminate in E(nd). All scores *S* are defined in one scoring matrix, where 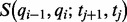 denotes the energy for stacking the base pair (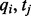) on (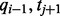). 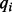 refers to the *i*th nucleotide in the query, 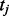 to the *j*th nucleotide in the target. Both are indexed in 5′- to 3′-direction from 1 to *m*, respectively, *n*. As two RNA strands interact running in opposite directions (antiparallel), target indices are decremented when query indices are incremented.

For each of the main states, we maintain one table (DP matrix) where 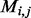 contains the maximum score of an interaction that ends ungapped at position *i* of the query and *j* in the target. Accordingly, *Bq* and *Bt* hold maximum scores for sub-alignments ending in a gap.

To lower memory usage from 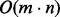, we split the algorithm in two steps. In the first step, RIsearch scans for possible interaction sites. By approximating loop energies with a linear (affine) model, only two rows of each DP matrix need to be stored. All scores for the current row only depend on the previous row, achieving a linear space requirement. During this phase, we store for each row the maximum score and its position in the query (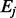 and 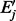). This results in a space complexity of 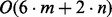 with *m* and *n* being the lengths of the query and target sequence, respectively. This approach introduces an ambiguity, as each position in the target can only be linked to one position in the query. If there are multiple sites within the query that bind to the same region of the target, the weaker interaction might be missed. In practice, however, this does not cause problems when the query is a short sequence. In the second step, all entries in *E* that exceed a given threshold are processed. For this, a region of 40 nt (or a user-specified amount) downstream of the identified positions is taken into account to compute the actual structure and free energy of the duplexes. In this way, RIsearch needs only marginally more time to identify suboptimal interactions.

This approach is similar to RNAplex. We reach a further simplification by (i) not using an extra state for interior loops, and (ii) also approximate small interior loops with the affine model instead of relying on the look-up tables. (iii) RNAplex seems to incorporate dangling end contributions even though this is not stated in their paper. Fewer states lead to a less complex recursion and other differences are due to algorithmic design (most notably our dinucleotide matrix). This also holds true for a comparison with BINDIGO that distinguishes different types of bulges and interior loops depending on their size and degree of asymmetry and includes terminal stacks, thus leading to a more complex recursion.

The RIsearch recursion is given as

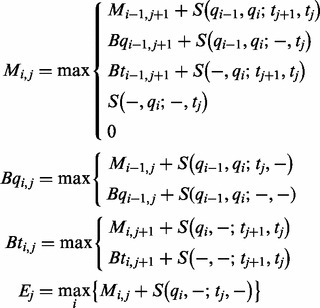



Entries in the *M*-state (ungapped) can come from (i) the *M*-state by extending the previous alignment with one residue on both strands, meaning either a stacking of a new pair, or the symmetric extension of an interior loop (or opening, closing an interior loop). The entries can (ii/iii) come from a dash (gap) in either sequence, reflecting the closing of a bulge or the closing or continuation of an asymmetric interior loop. (iv) A new alignment can be started and (v) 0 is given if this pair should not be part of the interaction. In the implementation, the latter two cases are merged into one, implicitly handled by the scoring matrix. All scores 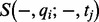 where 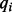 and 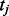 do not form a Watson–Crick or wobble base pair, and therefore should not start an alignment, are set to zero. Elements in *Bq* and *Bt* can either come from the *M*-state by opening a new ‘gap’ or from the same state by extending an existing ‘gap’. As mentioned before, the row maximum is stored in a one-dimensional array 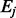 and the corresponding position *i* within the query in 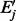. One possibility to allow a position in the target to be related to more than one position in the query is to make these arrays two-dimensional, giving room to store the second and third best interaction in 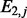 and 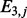.

### 2.2 Scoring matrix

**Fig. 2.**
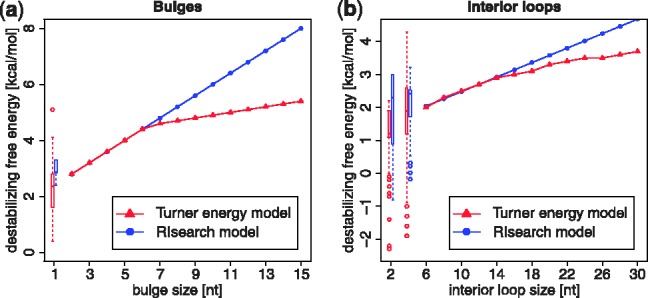
Approximated loop energies. In red, energies as given by Turner 2004 parameters. In blue, the linear approximation used in RIsearch. Values for small loops are given as box plots (RIsearch to the right). (**a**) Bulge loops: the affine gap model is exact for bulge sizes 2–6, and over-penalizes larger loops. (**b**) Interior loops: here symmetric case only, for asymmetric loops a penalty is added. Furthermore, parameters for AU/GU closure and terminal mismatch are applied where required in both schemes. Small symmetric internal loops (1 × 1 and 2 × 2) have tabulated free energy changes, here shown as box plots. Next to that, RIsearch approximations are plotted, including the aforementioned parameters

Values for the 36 × 36 scoring matrix (Supplementary Fig. S1) are derived from the Nearest Neighbor Database (NNDB) (Turner and Mathews, 2010). The NNDB contains parameters, determined from optical melting experiments, that allow prediction of free energy changes of the different RNA structural elements (stacked pairs, loops) and are widely used in software for RNA folding. It provides complete nearest-neighbor sets, including rules and parameter values, along with tutorials. Considering the current and previous position of the two sequences allows us to apply stacking energies for the base pairs. Bulge and interior loop energies usually contain a length-dependent term, but are here approximated by an affine model. Figure 2a shows that the affine ‘gap’ model is exact for bulge sizes 2–6, and over-penalizes larger loops. A single-nucleotide bulge in our model receives the bulge opening cost and possibly a penalty for terminal A–U or G–U pairs. This is not the case for the full energy model, in which the stacking energy of the enclosing base pair is awarded. The look-up tables for interior loops of sizes 1 × 1, 1 × 2 and 2 × 2 cannot be incorporated into our scoring scheme. Instead, all energies have to be approximated by an affine model with opening and extension penalties, as depicted in Figure 2b. Interior loops with >14 nucleotides are over-penalized.

We created two matrices, one based on the so-called Turner 1999 energy parameters (Mathews *et al.*, 1999) and one based on the Turner 2004 set (Mathews *et al.*, 2004). The latter is the default, but the user can choose either. Energy contributions of stacking and bulges are largely identical, the scoring differs for interior loops. Supplementary Table S1 shows the free energies of example duplexes as modeled by RIsearch and other methods.

There are some ambiguous cases in the scoring matrix, for example in 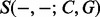. We cannot tell whether it is the extension of a bulge (0.4 kcal/mol) or the asymmetric extension of an already asymmetric interior loop (0.6 in T04 and 0.48 in T99). For this case, we decided to just assign the bulge loop extension penalty, as larger loops are already over-penalized.

To favor short stable interactions, the user can choose a per-nucleotide penalty [like in Tafer and Hofacker (2008)], which is then directly integrated in the scoring matrix.

### 2.3 Data and benchmarking

We benchmarked our method on several datasets containing simulated data and two real-life datasets of bacterial sRNA and human miRNA with their respective targets. Accuracy and runtime of RIsearch were compared with previous methods. We mainly focused on a comparison with RNAplex, because it belongs to the same class of algorithms and has already been benchmarked against a variety of other tools.

#### 2.3.1 Simulated data

This dataset consists of random sequences of different lengths (20–50 nt, in steps of 5 nt) covering a variety of GC-contents (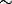
10, 30, 50, 70 and 90%). For each class, 1000 sequences were generated. First, the perfect complement was derived. Then, this hypothetical optimal binding partner was mutated stepwise as follows: A random position in the sequence was chosen to be substituted with 75% probability, deleted with 17% or a nucleotide inserted (8%). The number of repetitions is length dependent to ensure a wide range of Levenshtein distances (LDs) between the optimal and the mutated target, yielding 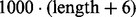 duplexes for each combination of length and GC-content.

For each of these duplexes, we calculate the minimum free energy (MFE) with different tools, namely DuplexFold from the RNAstructure package (Reuter and Mathews, 2010), which implements the NNDB 2004 rule set, RNAplex (Tafer *et al.*, 2011) and RNAcofold (Bernhart *et al.*, 2006b), which both use the Turner 1999 parameters and RIsearch with the 2004 matrix.

#### 2.3.2 Speed data

To benchmark time (and also memory) consumption, we use three different sets of sequences. Random data as well as genomic sequences of various lengths and different number of query sequences are contained in those. For details, see Section S2.1 in the Supplementary Material.

#### 2.3.3 sRNA data

This dataset comprises a total of 17 sRNA–mRNA interactions with experimentally verified binding site positions. It has been used before as a benchmark set by Busch *et al.* (2008), Chitsaz *et al.* (2009) and lately by Tafer *et al.* (2011) , from which sequences were taken. Query and target sequences are on average 147 nt and 179 nt long. The specific sRNA–mRNA interactions appear in the table in Section 3. Since RNAplex was previously benchmarked on a range of tools (Tafer and Hofacker, 2008; Tafer *et al.*, 2011), we only compare RIsearch (both parameter sets) with RNAplex (with and without taking into account accessibility). Accessibility profiles were computed by RNAplfold (Bernhart *et al.*, 2006a) with parameters as suggested by Tafer (RNAplfold -W 240 -L 160 -u 40 -O.)

For this benchmark we do not consider suboptimal duplexes initially, but only whether the first reported hit corresponds to the experimentally verified interaction site. We present the deviation between predicted and experimentally verified duplex boundaries as was also done by Tafer *et al.* (2011). To evaluate the performance, we additionally calculated the sensitivity (SEN, also called true-positive rate) and the precision [also called positive predictive value (PPV)] and the harmonic mean of both (also known as *F*-measure) as was also done by Kato *et al.* (2010) and Salari *et al.* (2010). The definitions are

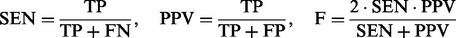



We counted true positives (TPs), false positives (FPs) and false negatives (FNs) by comparing the verified interacting base pairs with predicted ones. An alternative measure is the Matthews correlation coefficient (Matthews, 1975) , which in this case [since the number of true negatives (TNs) is orders of magnitudes higher than TP, FP and FN] reduces to the geometric mean of the sensitivity and the PPV: 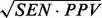 (Gorodkin *et al.*, 2001). For cases where the first prediction did not overlap the experimentally verified location, we then also computed suboptimal solutions.

#### 2.3.4 miRNA data

We examine a subset of human microRNAs causing mRNA repression from TarBase (Papadopoulos *et al.*, 2009). This dataset of 27 interactions has been used before to benchmark RNAplex (Tafer and Hofacker, 2008). The mature miRNA sequences from miRBase 17 (Kozomara and Griffiths-Jones, 2011) are used as query and the 3′-UTRs of the respective mRNAs from UCSC hg19 (average length 2294 nt) are used as target. The experimentally confirmed binding sites were collected from the original papers and mapped to the extracted sequences. For some miRNA–mRNA pairs, there is more than one verified interaction site. The majority have only one or two binding sites (13 and 9), few have up to four, the interactions including KRAS and NRAS forming the exception with eight and nine possible binding sites, respectively (Johnson *et al.*, 2005). The 27 miRNA/UTR pairs were scanned with RNAhybrid, RNAplex and RIsearch, allowing for suboptimal hits. For RIsearch, we used the 1999 energy parameters, as the other two methods also use them. An interaction counts as ‘recovered’ when the predicted target region overlaps any of the annotated binding sites. For interactions with more than one experimentally verified binding site, we considered the one ranked highest (according to their predicted free energy) for each method.

We used the same 27 interactions to test performance on large-scale screens. Instead of only the 3′-UTR sequence, we used the whole repeat-masked chromosome where the known target is located. We also included GUUGle as well as TargetScanS (Garcia *et al.*, 2011) and miRanda (Enright *et al.*, 2003), which both are specifically designed for miRNA target prediction. In this screen, we excluded RNAhybrid, because it requires to fill in the entire DP matrix several times to predict suboptimal duplexes, which makes it too slow for scanning whole chromosomes. For each of the five methods, we count the number of hits which have the same or better score than the best hit that overlaps an annotated target site. Two different measures were used to evaluate the methods. For the first measure, we ranked all methods individually for each miRNA–mRNA pair, where the method yielding the lowest hit count ranks first (using fractional ranking). Given *k* interactions, let 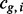 be the count and 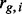 the rank of method *g* in the *i*th interaction. The rank product (Breitling *et al.*, 2004) is given as the geometric mean: 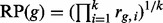. Because this is an ordinal measurement, we define a second measure, the relative hit score, which takes account for the degree of difference between the hit counts of the different methods. We first find the maximum count *N* for each interaction: 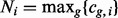 and define 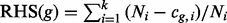.

To demonstrate the efficacy of RIsearch as a filter, we first use RIsearch (with free energy threshold of −11 kcal/mol) and apply TargetScanS and miRanda on the pre-filtered data. We measure the reduction in candidate regions compared with the raw results of the two miRNA target predictors. We compare this with the filter abilities of GUUGle (requiring a seed match of at least seven nucleotides) and a combination of the two.

## 3 RESULTS

### 3.1 General ranking of duplexes

**Fig. 3.**
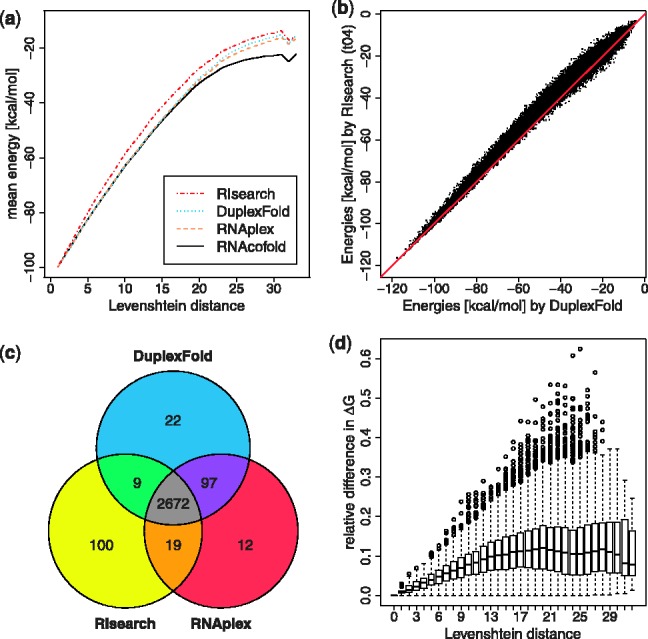
Accuracy on simulated data. Data shown here for length = 50 nt, GC-content = 50%. (**a**) Average of all computed MFEs given a certain LD as reported by the different tools. (**b**) Correlation of MFE values as returned by DuplexFold versus RIsearch. (**c**) Overlap of helices in the top 5% ranking predictions. (**d**) Relative difference in reported energies, computed as |(DuplexFold–RIsearch)/DuplexFold|. The boxes represent the interquartile range (IQR), from the first quartile to the third quartile, the band inside denotes the median. The whiskers extend to the most extreme data points within 1.5 IQR from the box. Outliers are shown as circles

To evaluate the accuracy of the scoring scheme, we created a set of random sequences for different combinations of length and GC-content as described above. We address not only score (energy) computations, but also the ranking by the various tools. In Figure 3a, we show how the different methods deviate in their scoring. RNAcofold was included here because it considers intramolecular base pairs resulting in lower energies for sequences that are further away from perfect complementarity. The energy deviation to the other tools increases with LD. RIsearch typically yields higher energies; however, the results are ranked similarly to the other methods. When comparing duplex free energies as computed by RIsearch and DuplexFold (Fig. 3b), we get a Pearson product-moment correlation coefficient *r* of 0.99 (*P*-value 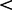
2.2e−16). The Spearman’s rank correlation coefficient 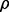 ranges between 0.98 and 0.99. Furthermore, when looking at the 5% highest ranking duplexes (those with the lowest 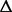
G), the overlap in candidates is quite substantial (Fig. 3c). Even though the free energies computed by RIsearch deviate from energies computed by a method using the full energy model (see comparison with DuplexFold in Fig. 3d), we have shown that the general ranking of the duplexes correlates well with the ranking by DuplexFold. See Supplementary Figure S2 for sequences of other lengths and GC-content.

### 3.2 Runtime and memory benchmark

As RNAplex has already been shown to be much faster than alternative methods, we only compare the performance of RIsearch to RNAplex on different datasets.

On a small dataset of 19 bacterial sRNAs and 100 target sequences (each 1200 nt long), RNAplex takes around 25 s to predict all optimal duplexes, whereas RIsearch only needs 9 s on a standard laptop (Intel C2D @2.53 GHz) (Supplementary Table S2).

To prove that whole-genome scans become feasible, we queried whole human chromosome 1 with one miRNA and got a speedup of 181-fold. With more sequences in the query, this drops drastically. From the data shown in Supplementary Table S3, it seems that RNAplex uses a substantial amount of time for the initialization. But also after correcting for that, RIsearch still shows a significant speedup.

To get a more complete picture, we generated random sequences with lengths of 10, 100, 1000 and 25 nt (the latter to represent ncRNAs of the type miRNA or small interfering RNA) as queries as well as target sequences in order of magnitude steps between 1000 and 1 GB. The speedup of RIsearch over RNAplex grows with decreasing query and increasing target lengths (Supplementary Table S4). The extreme speedups we see for large target sequences should be noted with caution, as they reflect the same initialization issue as mentioned above. This overhead in the initialization cannot be explained with more advanced options to RNAplex, parameters were chosen to yield fastest runtimes. The overhead might just be an implementation issue.

Overall, we observe a worst case speedup of around 2.4. Peak-memory consumption is typically reduced by a factor 1.44, i.e. RIsearch uses 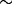
69% of the memory that RNAplex uses. With short target sequences (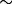
1000 nt), this drops to 43%. 

For all of these benchmarks the simple version of RNAplex was used, i.e*.* not taking into account accessibility. We also tested the version including accessibility profiles and found it substantially more resource demanding than the regular RNAplex, which itself in its current implementation is considerably slower than RIsearch (Supplementary Section S2.2).

### 3.3 Locating sRNA interaction sites

The precision of RIsearch (with 99 and 04 Turner parameters) was compared with RNAplex (with and without accessibility) on a real-life dataset of 17 bacterial sRNA–mRNA interactions. Although RNAplex-a (with accessibility) predicts 16 interactions that overlap the known binding sites, RIsearch with both energy parameter sets and RNAplex-c (without accessibility) each recover 12 of the known interaction sites (Table 1). There are two cases (GcvB-STM4351 and MicC-ompC) where each of those three methods predicts the same energetically more stable interaction than the annotated one. When measuring the amount of overlap of predicted and annotated base pairs (Supplementary Table S5), RNAplex-a performs best in all measures, with an average sensitivity and PPV (0.787 and 0.736) higher than RIsearch04 (0.656 and 0.641) and the other methods (ranging behind), because it only misses one interaction, not five. However, if suboptimal solutions are additionally taken into account, RIsearch04 has a better average sensitivity of 0.919, compared with 0.846 and 0.917 for RNAplex with and without accessibility, respectively. RIsearch (with its default scoring matrix) then also outperforms RNAplex in terms of PPV (RIsearch04: 0.898, RNAplex-a: 0.785 and RNAplex-c: 0.821), *F*-measure and Matthews correlation coefficient. In all measurements, the 2004 Turner parameters lead to a higher prediction accuracy than the 1999 parameters in RIsearch.

**Table 1.** Predicted sRNA target sites

Pair		Binding site literature		Positions RNAplex-a		Positions RNAplex-c 30		Positions RIsearch t99		Positions RIsearch t04	
sRNA	mRNA	sRNA	mRNA	sRNA	mRNA	sRNA	mRNA	sRNA	mRNA	sRNA	mRNA
GcvB	gltI	66; 77	31; 43	65; 76	32; 43	34; 74	33; 69	64; 102	12; 44	35; 66	128; 153
GcvB	argT	75; 91	89; 104	72; 90	90; 107	95; 125	168; 196	91; 124	169; 206	75; 91	89; 104
GcvB	dppA	65; 83	133; 150	57; 92	121; 157	56; 93	120; 158	57; 92	121; 157	57; 92	121; 157
GcvB	livJ	63; 87	59; 82	63; 87	59; 82	62; 88	58; 83	63; 87	59; 82	63; 87	59; 82
GcvB	livK	68; 77	165; 177	65; 90	150; 177	117; 126	240; 249	65; 97	146; 177	118; 125	241; 248
GcvB	oppA	65; 90	155; 179	65; 89	155; 178	64; 90	154; 179	65; 89	155; 178	65; 89	155; 178
GcvB	STM4351	70; 79	44; 52	62; 87	33; 58	35; 72	91; 124	30; 65	97; 131	30; 65	97; 131
MicA	lamB	8; 36	122; 148	5; 21	1; 16	57; 68	154; 165	8; 36	122; 148	8; 36	122; 148
MicA	ompA	8; 24	113; 128	8; 24	113; 128	7; 25	112; 129	8; 24	113; 128	8; 24	113; 128
DsrA	rpoS	8; 36	10; 38	21; 40	7; 25	9; 41	6; 37	10; 40	7; 36	10; 40	7; 36
RprA	rpoS	33; 62	16; 39	40; 71	1; 32	32; 46	26; 40	33; 45	27; 39	33; 45	27; 39
IstR	tisA	65; 87	57; 79	65; 87	57; 79	64; 93	50; 80	65; 92	51; 79	65; 92	51; 79
MicC	ompC	1; 30	93; 119	1; 16	104; 119	40; 66	71; 93	41; 65	72; 92	41; 65	72; 92
MicF	ompF	1; 33	100; 125	1; 28	105; 125	1; 34	99; 126	27; 32	68; 73	1; 33	100; 125
RyhB	sdhD	9; 50	89; 128	19; 41	98; 118	8; 42	97; 129	7; 41	98; 127	7; 41	98; 127
RyhB	sodB	38; 46	52; 60	38; 65	38; 60	37; 50	49; 61	38; 46	52; 60	38; 46	52; 60
SgrS	ptsG	157; 187	76; 107	168; 187	76; 95	167; 188	75; 96	7; 19	38; 53	7; 19	38; 53

For each sRNA–mRNA interaction, we report the binding site (begin and end positions in both sequences) as given in the literature and as predicted by the four methods. All methods searched only for the single best-scoring interaction. RNAplex-a uses pre-computed accessibility profiles and only misses one interaction (in bold and gray text). RIsearch (with 99 and 04 Turner parameters) and RNAplex-c do not take into account accessibility information and instead use a fixed per-nucleotide penalty of 0.3 kcal/mol. These three methods miss five interactions each, though different ones.

### 3.4 Locating miRNA target sites in genomic sequence

We also compared the recovery rates of RIsearch, RNAplex and RNAhybrid for 27 human miRNA–target UTR duplexes. Predicted binding sites were ranked according to their free energy and we report the highest ranking prediction that overlaps an annotated binding site in Table 2, as it has been done by Tafer and Hofacker (2008). For 23 of the interactions, the three methods report the same binding site as the highest scoring true-positive hit. For example, TarBase contains four possible binding sites for the let-7e miRNA in the 3′-UTR of SMC1A mRNA. They constitute the top ranking candidates of all the methods tested here, but in different order. The interaction that has been experimentally verified (Kiriakidou *et al.*, 2004) is ranked first by RIsearch and second by the other methods. In this benchmark, RNAhybrid performs best, known target sites are ranked higher and predicted more accurately in position. RNAplex is slightly better than RIsearch in ranking the real interactions (on average 1.92 compared with 2.07), while RIsearch predictions are generally closer to the verified binding site. The frequent one-nucleotide deviation in position could be an artifact of the different handling of dangling ends and terminal mismatches.

**Table 2.** Predicted miRNA target sites

mRNA	miRNA	RNAhybrid ( 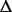 *G r p*)	RNAplex ( 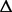 *G r p*)	RIsearch ( 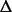 *G r p*)
AGTR1	miR-155	−20.90 1 11	−14.37 1 11	−14.06 1 9
BCL2	miR-16	−24.10 1 1	−18.90 2 1	−18.69 1 1
SLC7A1	miR-122	−29.00 1 2	−23.80 1 2	−23.04 1 4
TPPP3	miR-16	−26.00 2 0	−20.80 2 1	−19.95 1 1
CLOCK	miR-141	−22.10 1 0	−16.40 1 0	−15.37 1 7
CXCL12	miR-23a	−25.90 1 38	−19.80 2 37	−18.00 2 38
CYP1B1	miR-27b	−33.60 1 1	−28.20 1 1	−26.91 1 1
E2F3	miR-34a	−25.10 2 10	−19.10 2 10	−18.31 2 1
EZH2	miR-101	−22.40 1 1	−16.90 1 1	−15.65 1 1
PARP8	miR-145	−27.40 1 3	−21.80 1 3	−20.00 1 1
FSTL1	miR-206	−23.20 3 0	−18.40 4 0	−15.87 6 2
GJA1	miR-1	−20.60 1 1	−14.30 2 1	−12.76 2 12
GJA1	miR-206	−21.00 4 11	−15.03 6 11	−12.55 8 0
HAND2	miR-1	−18.10 1 1	−12.20 1 1	−9.61 2 5
HOXA1	miR-10a	−23.30 1 14	−15.93 4 12	−12.71 4 13
KIT	miR-221	−23.40 3 0	−17.70 3 0	−15.18 2 2
KIT	miR-222	−23.70 3 58	−18.60 4 56	−15.38 5 56
KRAS	let-7a	−21.30 5 2	−16.30 3 3	−15.03 3 3
LIN28A	let-7b	−33.50 1 1	−27.40 1 1	−25.20 1 5
MAPK14	miR-24	−32.20 1 1	−27.10 1 0	−25.98 1 1
MYCN	miR-101	−20.70 1 1	−13.85 2 16	−12.19 1 1
NRAS	let-7a	−21.60 4 6	−17.70 2 22	−13.96 4 8
PTEN	miR-19a	−23.20 1 1	−17.70 1 1	−16.74 1 1
ARHGAP32	miR-132	−25.10 1 3	−18.80 1 3	−18.55 1 1
SMC1A	let-7e	−27.70 1 1	−22.20 1 1	−21.49 1 2
TMSB4X	miR-1	−21.90 1 1	−16.90 1 1	−16.76 1 1
TPM1	miR-21	−20.00 1 13	−15.00 1 8	−13.44 1 7
Average rank and position		1.67/6.74	1.93/7.56	2.07/6.81

Column 1: HGNC [HUGO (Human Genome Organization) Gene Nomenclature Committee] symbol, column 2: miRNA ID (all human), columns 3–5: Results shown for each of the tools include the 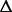
*G* (kcal/mol) of the interaction, its rank (*r*) within all predictions and the deviation [nt] of the predicted target position *pred* from the reported one *lit* (*p*) with *p *= 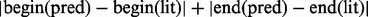. Note, that for RNAhybrid the energies are usually lower, because the initiation energy of 4.1 kcal/mol is not included. The last row contains the average of the rank and the deviation in position.

When screening the whole chromosome, we observe very different levels of specificity (Supplementary Table S6). TargetScanS fails to find 2 out of the 27 interactions, because it requires a perfect seed match that is not present in those target sites. With default parameters, miRanda misses three interactions. We have not tried any other parameter setting. GUUGle alone performs worst (in terms of RP and RHS), but when combined with RIsearch or RNAplex, respectively, we get an miRNA target predictor comparable to the specialized methods. In this combination, RIsearch scores best in RHS and slightly behind the two specialized methods in RP.

When applying this combination as pre-filter for TargetScanS and miRanda, we achieve on average a reduction of candidates by around 37% for both tools. RIsearch without the GUUGle-prefilter accounts for an average reduction of 27% for miRanda candidates and 35% for TargetScanS, in some cases of up to 70% (Supplementary Table S7). The degree of reduction seems dependent on the GC-content of the miRNA (see Supplementary Material, page 11).

The prediction of thousands of potential miRNA targets is in agreement with a recent hypothesis of miRNA response elements connecting mRNAs, transcribed pseudogenes and long ncRNAs in large-scale regulatory networks (Salmena *et al.*, 2011).

### 3.5 Filtering using RIsearch

**Fig. 4.**
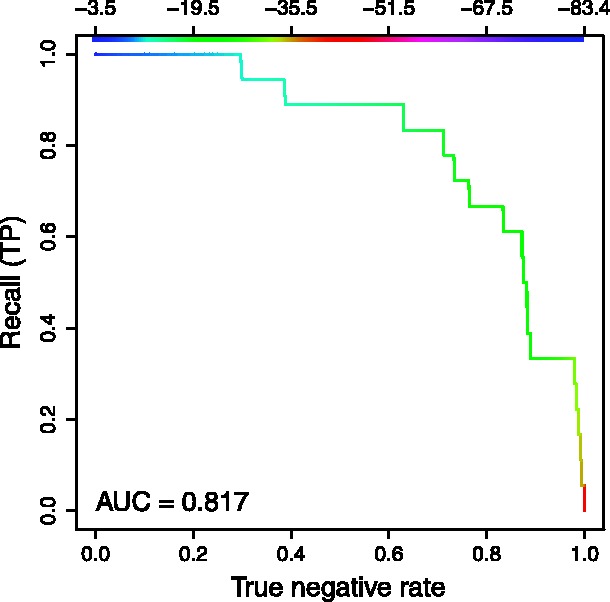
RIsearch as filter for bacterial sRNA–mRNA interactions. The color key refers to RIsearch energy cutoffs. TNR (or specificity) is synonymous with the search space reduction we can achieve with different cutoffs. Recall (or sensitivity, TPR) shows how many of the known interactions we retain

Here, we illustrate how RIsearch can be used as filter for the more complex algorithms, such as IntaRNA or RNAup. We extracted the sequences from *Escherichia coli* and *Salmonella typhimurium* according to the IntaRNA paper (Busch *et al.*, 2008). In Figure 4, we show a receiver-operating characteristic curve of the recall against search space reduction given different energy cutoffs, with an area under the curve of 0.817. It shows that with a rather conservative cutoff of −10 kcal/mol, we can already filter out 16.1% of the candidates. With −11.5 kcal/mol, we still retain all true targets, while reducing the search space by 27.5%. Considering that IntaRNA uses around 9 h 40 min (and RNAup even 18 h 50 min) to compute all 47.726 duplexes, while RIsearch takes <18 s, this shows how genome-wide searches can be speeded up.

## 4 DISCUSSION

RIsearch is a fast method to search for near-complementary base pairing in genomic sequence by using a simplified energy model. In a runtime benchmark, we show that RIsearch is at a minimum a factor of 2.4 faster than RNAplex, the currently fastest method for predicting near-complementary duplexes and also has a lower memory consumption. Remarkably, our simplified model gives good energy estimates for complementary duplexes interspersed with small bulges and interior loops. Even though RIsearch systematically computes an energy differing by a small factor compared with the full Turner energy model, the reported energies strongly correlate (*r* = 0.99) with the energies computed by the full model in DuplexFold. When ranking random duplexes by their predicted energies, RIsearch shows on average an overlap of 94% with DuplexFold and RNAplex within the highest ranking duplexes.

In our evaluation of prediction accuracy on the sRNA–mRNA and miRNA–mRNA datasets, we show that RIsearch achieves a similar or better sensitivity and precision for predicted base pairs as other compared methods. However, considering the accessibility of binding sites with RNAplex can increase the recovery rate of the verified sRNA–mRNA interactions. Other approaches, such as IntaRNA and RNAup, also account for accessibility by computing intramolecular base pair probabilities in both sequences. This, however, comes at the expense of runtime. In contrast, the objective of RIsearch is the fast search for potential RNA–RNA duplexes. One application is pre-filtering in genome-wide screens. When RIsearch is used as a pre-filter for specialized miRNA target predictors, such as miRanda and TargetScanS, the number of target site candidates can be significantly reduced, which in turn results in a better precision of the miRNA target prediction.

Problems with developing methods that should be applied on a genome-wide scale include reliable testing. We face a lack of experimentally verified interactions. For many miRNAs for example, target genes have been identified, but the actual binding site positions within their 3′-UTR are unknown (Lindow and Gorodkin, 2007). Even for known target sites, the extent of the interactions is not clear, for examples of bacterial sRNA target sites, see Sharma *et al.* (2007). Even though we could benchmark on a limited dataset, benchmarking the accuracy of genome-wide searches for RNA–RNA interactions is currently hard given the limited amount of known interactions. In particular, it is not possible to reliably calculate the false-discovery rate unless follow-up experiments are carried out. Another factor is the estimation of *P*-values, which requires a background model for RNA–RNA interaction to distinguish true positives from random hits. This, however, depends on incorporating reliable shuffling schemes, e.g. based on dinucleotide composition, similar to those for *de novo* prediction of ncRNA genes (Gorodkin and Hofacker, 2011).

RIsearch was developed as a tool, which can conduct a fast initial screen in genomic sequence and aid in the overall goal of mapping all potential RNA–RNA interactions in, e.g. the human genome. However, it is beyond the scope of this work to set up such a pipeline, which most likely involves additional methods, taking the full energy model into account, as well as the development of a framework for computing *P*-values. The estimation of *P*-values by TargetRNA points to a direction for this. For further future directions, prediction of RNA–RNA interactions could be combined with high-throughput experimental data, such as done for RNA structure prediction (Deigan *et al.*, 2009; Kertesz *et al.*, 2010; Underwood *et al.*, 2010).

The introduced simplifications will make a hardware implementation of the algorithm, e.g. with a field-programmable gate array, more feasible. Hardware accelerated versions of the Smith–Waterman algorithm have been shown to be magnitudes faster than traditional software implementations (Li *et al.*, 2007).

## Supplementary Material

Supplementary Data

Supplementary Data
