# Supplementary material for: RIsearch: fast RNA–RNA interaction search using a simplified nearest-neighbor energy model
Source: Bioinformatics. 2012 Aug 24;28(21):2738–46. doi: 10.1093/bioinformatics/bts519 (PMC3476332; doi:10.1093/bioinformatics/bts519)
Supplement: Supplementary Data [file supp_28_21_2738__index.html]

RIsearch: fast RNA–RNA interaction search using a simplified nearest-neighbor energy model — Supplementary Data 

# RIsearch: fast RNA–RNA interaction search using a simplified nearest-neighbor energy model

## Supplementary Data

files

**Files in this Data Supplement:**

- Supplementary Data - pdf file
