# Supplementary material for: RIsearch: fast RNA–RNA interaction search using a simplified nearest-neighbor energy model
Source: Bioinformatics. 2012 Aug 24;28(21):2738–46. doi: 10.1093/bioinformatics/bts519 (PMC3476332; doi:10.1093/bioinformatics/bts519)
Supplement: Supplementary Data [file supp_bts519_supplement.pdf]

# Supplementary Materials

## RIsearch: Fast RNA-RNA Interaction Search using a Simplified Nearest-Neighbor Energy Model

Anne Wenzel, Erdinç Akbaşlı and Jan Gorodkin

August 10, 2012

## 1 The RIsearch scoring scheme

| | AA | AC | AG | AU | AN | A– | CA | CC | CG | CU | CN | C– | GA | GC | GG | GU | GN | G– | UA | UC | UG | UU | UN | U– | NA | NC | NG | NU | NN | N– | –A | –C | –G | –U | –N | – – |
|---|---|---|---|---|---|---|---|---|---|---|---|---|---|---|---|---|---|---|---|---|---|---|---|---|---|---|---|---|---|---|---|---|---|---|---|---|
| AA | -18 | -18 | -18 | -70 | -18 | -69 | -18 | -18 | -18 | -70 | -18 | -69 | -18 | -18 | -18 | 30 | -18 | -69 | -246 | -246 | -166 | 90 | -246 | -285 | -18 | -18 | -18 | -70 | -18 | -69 | -18 | -18 | -18 | -45 | -18 | -40 |
| AC | -18 | -18 | 0 | -18 | -18 | -69 | -18 | -18 | 0 | -18 | -18 | -69 | -18 | -18 | 100 | -18 | -18 | -69 | -246 | -246 | 220 | -246 | -246 | -285 | -18 | -18 | 0 | -18 | -18 | -69 | -18 | -18 | 0 | -18 | -18 | -40 |
| AG | -18 | 0 | -18 | -70 | -18 | -69 | -18 | 0 | -18 | -70 | -18 | -69 | -18 | 100 | -18 | 30 | -18 | -69 | -146 | 210 | -126 | 60 | -246 | -285 | -18 | 0 | -18 | -70 | -18 | -69 | -18 | 0 | -18 | -45 | -18 | -40 |
| AU | -70 | -18 | -70 | -18 | -18 | -69 | -70 | -18 | -70 | -18 | -18 | -69 | 30 | -18 | 30 | -18 | -18 | -69 | 110 | -246 | 140 | -176 | -246 | -285 | -70 | -18 | -70 | -18 | -18 | -69 | -45 | -18 | -45 | -18 | -18 | -40 |
| AN | -18 | -18 | -18 | -18 | -18 | -69 | -18 | -18 | -18 | -18 | -18 | -69 | -18 | -18 | -18 | -18 | -18 | -69 | -246 | -246 | -246 | -246 | -246 | -285 | -18 | -18 | -18 | -18 | -18 | -69 | -18 | -18 | -18 | -18 | -18 | -40 |
| A– | -69 | -69 | -69 | -69 | -69 | -∞ | -69 | -69 | -69 | -69 | -69 | -∞ | -69 | -69 | -69 | -69 | -69 | -∞ | -285 | -285 | -285 | -285 | -285 | -45 | -69 | -69 | -69 | -69 | -69 | -∞ | * | * | * | * | * | * |
| CA | -18 | -18 | -18 | -70 | -18 | -69 | -18 | -18 | -18 | -70 | -18 | -69 | -176 | -176 | -96 | 210 | -176 | -240 | -18 | -18 | -18 | -70 | -18 | -69 | -18 | -18 | -18 | -70 | -18 | -69 | -18 | -18 | -18 | -45 | -18 | -40 |
| CC | -18 | -18 | 0 | -18 | -18 | -69 | -18 | -18 | 0 | -18 | -18 | -69 | -176 | -176 | 330 | -176 | -176 | -240 | -18 | -18 | 0 | -18 | -18 | -69 | -18 | -18 | 0 | -18 | -18 | -69 | -18 | -18 | 0 | -18 | -18 | -40 |
| CG | -18 | 0 | -18 | -70 | -18 | -69 | -18 | 0 | -18 | -70 | -18 | -69 | -76 | 240 | -56 | 140 | -176 | -240 | -18 | 0 | -18 | -70 | -18 | -69 | -18 | 0 | -18 | -70 | -18 | -69 | -18 | 0 | -18 | -45 | -18 | -40 |
| CU | -70 | -18 | -70 | -18 | -18 | -69 | -70 | -18 | -70 | -18 | -18 | -69 | 210 | -176 | 210 | -106 | -176 | -240 | -70 | -18 | -70 | -18 | -18 | -69 | -70 | -18 | -70 | -18 | -18 | -69 | -45 | -18 | -45 | -18 | -18 | -40 |
| CN | -18 | -18 | -18 | -18 | -18 | -69 | -18 | -18 | -18 | -18 | -18 | -69 | -176 | -176 | -176 | -176 | -176 | -240 | -18 | -18 | -18 | -18 | -18 | -69 | -18 | -18 | -18 | -18 | -18 | -69 | -18 | -18 | -18 | -18 | -18 | -40 |
| C– | -69 | -69 | -69 | -69 | -69 | -∞ | -69 | -69 | -69 | -69 | -69 | -∞ | -240 | -240 | -240 | -240 | -240 | 0 | -69 | -69 | -69 | -69 | -69 | -∞ | -69 | -69 | -69 | -69 | -69 | -∞ | * | * | * | * | * | * |
| GA | -18 | -18 | -18 | 10 | -18 | -69 | -176 | -176 | -96 | 240 | -176 | -240 | -18 | -18 | -18 | 50 | -18 | -69 | -246 | -246 | -166 | 130 | -246 | -285 | -18 | -18 | -18 | -70 | -18 | -69 | -18 | -18 | -18 | -45 | -18 | -40 |
| GC | -18 | -18 | 80 | -18 | -18 | -69 | -176 | -176 | 340 | -176 | -176 | -240 | -18 | -18 | 120 | -18 | -18 | -69 | -246 | -246 | 250 | -246 | -246 | -285 | -18 | -18 | 0 | -18 | -18 | -69 | -18 | -18 | 0 | -18 | -18 | -40 |
| GG | -18 | 80 | -18 | 10 | -18 | -69 | -76 | 330 | -56 | 150 | -176 | -240 | -18 | 120 | -18 | 50 | -18 | -69 | -146 | 210 | -126 | 50 | -246 | -285 | -18 | 0 | -18 | -70 | -18 | -69 | -18 | 0 | -18 | -45 | -18 | -40 |
| GU | 10 | -18 | 10 | -18 | -18 | -69 | 220 | -176 | 250 | -106 | -176 | -240 | 50 | -18 | 50 | -18 | -18 | -69 | 140 | -246 | -130 | -176 | -246 | -285 | -70 | -18 | -70 | -18 | -18 | -69 | -45 | -18 | -45 | -18 | -18 | -40 |
| GN | -18 | -18 | -18 | -18 | -18 | -69 | -176 | -176 | -176 | -176 | -176 | -240 | -18 | -18 | -18 | -18 | -18 | -69 | -246 | -246 | -246 | -246 | -246 | -285 | -18 | -18 | -18 | -18 | -18 | -69 | -18 | -18 | -18 | -18 | -18 | -40 |
| G– | -69 | -69 | -69 | -69 | -69 | -∞ | -240 | -240 | -240 | -240 | -240 | 0 | -69 | -69 | -69 | -69 | -69 | -∞ | -285 | -285 | -285 | -285 | -285 | -45 | -69 | -69 | -69 | -69 | -69 | -∞ | * | * | * | * | * | * |
| UA | -246 | -246 | -166 | 130 | -246 | -285 | -18 | -18 | -18 | -70 | -18 | -69 | -246 | -246 | -166 | 100 | -246 | -285 | -18 | -18 | -18 | 0 | -18 | -69 | -18 | -18 | -18 | -70 | -18 | -69 | -18 | -18 | -18 | -45 | -18 | -40 |
| UC | -246 | -246 | 240 | -246 | -246 | -285 | -18 | -18 | 0 | -18 | -18 | -69 | -246 | -246 | 150 | -246 | -246 | -285 | -18 | -18 | 70 | -18 | -18 | -69 | -18 | -18 | 0 | -18 | -18 | -69 | -18 | -18 | 0 | -18 | -18 | -40 |
| UG | -146 | 210 | -126 | 100 | -246 | -285 | -18 | 0 | -18 | -70 | -18 | -69 | -146 | 140 | -126 | -30 | -246 | -285 | -18 | 70 | -18 | 0 | -18 | -69 | -18 | 0 | -18 | -70 | -18 | -69 | -18 | 0 | -18 | -45 | -18 | -40 |
| UU | 90 | -246 | 130 | -176 | -246 | -285 | -70 | -18 | -70 | -18 | -18 | -69 | 60 | -246 | 50 | -176 | -246 | -285 | 0 | -18 | 0 | -18 | -18 | -69 | -70 | -18 | -70 | -18 | -18 | -69 | -45 | -18 | -45 | -18 | -18 | -40 |
| UN | -246 | -246 | -246 | -246 | -246 | -285 | -18 | -18 | -18 | -18 | -18 | -69 | -246 | -246 | -246 | -246 | -246 | -285 | -18 | -18 | -18 | -18 | -18 | -69 | -18 | -18 | -18 | -18 | -18 | -69 | -18 | -18 | -18 | -18 | -18 | -40 |
| U– | -285 | -285 | -285 | -285 | -285 | -45 | -69 | -69 | -69 | -69 | -69 | -∞ | -285 | -285 | -285 | -285 | -285 | -45 | -69 | -69 | -69 | -69 | -69 | -∞ | -69 | -69 | -69 | -69 | -69 | -∞ | * | * | * | * | * | * |
| NA | -18 | -18 | -18 | -70 | -18 | -69 | -18 | -18 | -18 | -70 | -18 | -69 | -18 | -18 | -18 | -70 | -18 | -69 | -18 | -18 | -18 | -70 | -18 | -69 | -18 | -18 | -18 | -70 | -18 | -69 | -18 | -18 | -18 | -45 | -18 | -40 |
| NC | -18 | -18 | 0 | -18 | -18 | -69 | -18 | -18 | 0 | -18 | -18 | -69 | -18 | -18 | 0 | -18 | -18 | -69 | -18 | -18 | 0 | -18 | -18 | -69 | -18 | -18 | 0 | -18 | -18 | -69 | -18 | -18 | 0 | -18 | -18 | -40 |
| NG | -18 | 0 | -18 | -70 | -18 | -69 | -18 | 0 | -18 | -70 | -18 | -69 | -18 | 0 | -18 | -70 | -18 | -69 | -18 | 0 | -18 | -70 | -18 | -69 | -18 | 0 | -18 | -70 | -18 | -69 | -18 | 0 | -18 | -45 | -18 | -40 |
| NU | -70 | -18 | -70 | -18 | -18 | -69 | -70 | -18 | -70 | -18 | -18 | -69 | -70 | -18 | -70 | -18 | -18 | -69 | -70 | -18 | -70 | -18 | -18 | -69 | -70 | -18 | -70 | -18 | -18 | -69 | -45 | -18 | -45 | -18 | -18 | -40 |
| NN | -18 | -18 | -18 | -18 | -18 | -69 | -18 | -18 | -18 | -18 | -18 | -69 | -18 | -18 | -18 | -18 | -18 | -69 | -18 | -18 | -18 | -18 | -18 | -69 | -18 | -18 | -18 | -18 | -18 | -69 | -18 | -18 | -18 | -18 | -18 | -40 |
| N– | -69 | -69 | -69 | -69 | -69 | -∞ | -69 | -69 | -69 | -69 | -69 | -∞ | -69 | -69 | -69 | -69 | -69 | -∞ | -69 | -69 | -69 | -69 | -69 | -∞ | -69 | -69 | -69 | -69 | -69 | -∞ | * | * | * | * | * | * |
| –A | -18 | -18 | -18 | -45 | -18 | * | -18 | -18 | -18 | -45 | -18 | * | -18 | -18 | -18 | -45 | -18 | * | -18 | -18 | -18 | -45 | -18 | * | -18 | -18 | -18 | -45 | -18 | * | 0 | 0 | 0 | 105 | 0 | * |
| –C | -18 | -18 | 0 | -18 | -18 | * | -18 | -18 | 0 | -18 | -18 | * | -18 | -18 | 0 | -18 | -18 | * | -18 | -18 | 0 | -18 | -18 | * | -18 | -18 | 0 | -18 | -18 | * | 0 | 0 | 150 | 0 | 0 | * |
| –G | -18 | 0 | -18 | -45 | -18 | * | -18 | 0 | -18 | -45 | -18 | * | -18 | 0 | -18 | -45 | -18 | * | -18 | 0 | -18 | -45 | -18 | * | -18 | 0 | -18 | -45 | -18 | * | 0 | 150 | 0 | 105 | 0 | * |
| –U | -45 | -18 | -45 | -18 | -18 | * | -45 | -18 | -45 | -18 | -18 | * | -45 | -18 | -45 | -18 | -18 | * | -45 | -18 | -45 | -18 | -18 | * | -45 | -18 | -45 | -18 | -18 | * | 105 | 0 | 105 | 0 | 0 | * |
| –N | -18 | -18 | -18 | -18 | -18 | * | -18 | -18 | -18 | -18 | -18 | * | -18 | -18 | -18 | -18 | -18 | * | -18 | -18 | -18 | -18 | -18 | * | -18 | -18 | -18 | -18 | -18 | * | 0 | 0 | 0 | 0 | 0 | * |
| – – | -40 | -40 | -40 | -40 | -40 | * | -40 | -40 | -40 | -40 | -40 | * | -40 | -40 | -40 | -40 | -40 | * | -40 | -40 | -40 | -40 | -40 | * | -40 | -40 | -40 | -40 | -40 | * | * | * | * | * | * | -∞ |

stacked pairs
end duplex
begin duplex
bulge opening
bulge extension
bulge closing
int. loop opening
int. loop closing
int. loop extension symmetric
int. loop extension asymmetric
int. loop extension after asym.
forbidden (see below)
not begin duplex with mismatch

Figure 1: The scoring matrix. Free energy contributions in kcal/mol are derived from Turner's nearest neighbor parameters [1], and multiplied by -100 to build the score. This has the advantage that we get integers and can maximize over all possible local 'alignments'. Letters in the first column denote consecutive nucleotides in the query sequence (5' to 3'). Letters in the first row refer to the target sequence (3' to 5'). Reading example: The energy contribution of stacking a `CG` pair onto an `AU` pair (-2.2 kcal/mol) can be found in the row '`AC`' and column '`UG`', resulting in 220. $-\infty$ prevents alignments ending with a mismatch, it is set to -2000 in the implementation. $*$ denotes cases that the algorithm itself forbids, such as having a gap in one sequence followed by a gap in the other (insertion after deletion). It is set to an arbitrary value, as it is never read.

<table>
<tr><th></th><th>5'AGCGCU<br>||||||<br>3'UCGCGA</th><th>5'GCACG<br>|||||<br>3'CGUGC</th><th>5'GGUCGUGU<br>|::|::|:<br>3'CUGGUGCG</th><th>5'GGCCGUC<br>||| |||<br>3'CCG-CAG</th><th>5'GCGAACUGCACG<br>||||   |||||<br>3'CGCU---CGUGC</th><th>5'CAGACG<br>||  ||<br>3'GUAGGC</th><th>5'GCGA--G--CACG<br>||||     ||||<br>3'CGCUGAAAGGUGC</th><th>5'CCAG-ACGAG<br>|||  ||||<br>3'GGUGAGGCUC</th></tr>
<tr><td>TurnerNNDB99</td><td>-7.94</td><td>-6.04</td><td>-3.62</td><td>-5.74</td><td>-10.52</td><td>-1.50</td><td>-6.18</td><td>-6.24</td></tr>
<tr><td>RNAduplex*</td><td>-8.30</td><td>-6.00</td><td>-3.50</td><td>-5.80</td><td>-10.50</td><td>-1.50</td><td>-6.10</td><td>-6.30</td></tr>
<tr><td>RNAplex</td><td>-8.30</td><td>-6.00</td><td>-3.50</td><td>-5.80</td><td>-10.50</td><td>-1.50</td><td>-4.60</td><td>-6.30</td></tr>
<tr><td>RNAhybrid [2]</td><td>-13.40</td><td>-10.10</td><td>-6.30</td><td>-9.90</td><td>-15.10</td><td>-4.20</td><td>-11.00</td><td>-10.60</td></tr>
<tr><td>RIsearch t99</td><td>-8.41</td><td>-6.01</td><td>-1.16</td><td>-4.81</td><td>-10.56</td><td>-0.20</td><td>-6.84</td><td>-6.30</td></tr>
<tr><td>TurnerNNDB04</td><td>-7.94</td><td>-6.04</td><td>-3.62</td><td>-7.06</td><td>-10.52</td><td>-1.50</td><td>-5.65</td><td>-6.77</td></tr>
<tr><td>DuplexFold [3]</td><td>-8.30</td><td>-6.00</td><td>-1.10</td><td>-7.10</td><td>-10.50</td><td>-1.50</td><td>-5.70</td><td>-6.80</td></tr>
<tr><td>RIsearch t04</td><td>-8.41</td><td>-6.01</td><td>-1.16</td><td>-4.81</td><td>-10.56</td><td>0.13</td><td>-7.80</td><td>-7.18</td></tr>
</table>

Table 1: Possibilities and limitations of the scoring scheme. The table shows the free energies in kcal/mol as computed by different methods for the specified duplexes. Example duplexes with corresponding energies were retrieved from `http://rna.urmc.rochester.edu/NNDB/tutorials.html` (bulge and interior loops had to be extended with generic WC-helices to be predicted by the different tools). Upper part of table: Tools based on Turner 1999 parameters, lower part: tools based on 2004 parameter set. The top rows ('TurnerNNDBxx') should be considered as reference, methods deviate from this because of rounding and because some of the more complex rules are not implemented in the algorithms. (*) The same predictions as given by `RNAduplex`, were also reported by `RNAcofold` [4], `BINDIGO` [5] (web server only), and `PairFold` (web server) [6]. Apparently, `RNAhybrid` does not apply the intermolecular initiation energy that is typically given with +4.1 kcal/mol, thus yielding much lower energies.
The interaction in the first column is a self-complementary duplex, but none of the methods seems to implement the suggested symmetry correction. The third duplex shows the stack of `GU` followed by `UG` in two different contexts, stabilizing and destabilizing. As we only look at one previous position we cannot incorporate these scores. Similarly, look-up tables for the 2×2 internal loop in duplex number six are not used by `RIsearch`.

Figure 2: Accuracy on simulated data (next page). Variations of Figure 3 from the main paper for sequences of different length and GC-content. The Pearson product-moment correlation coefficient $r$ and Spearman's rank correlation coefficient $\rho$ are given in panel (b).

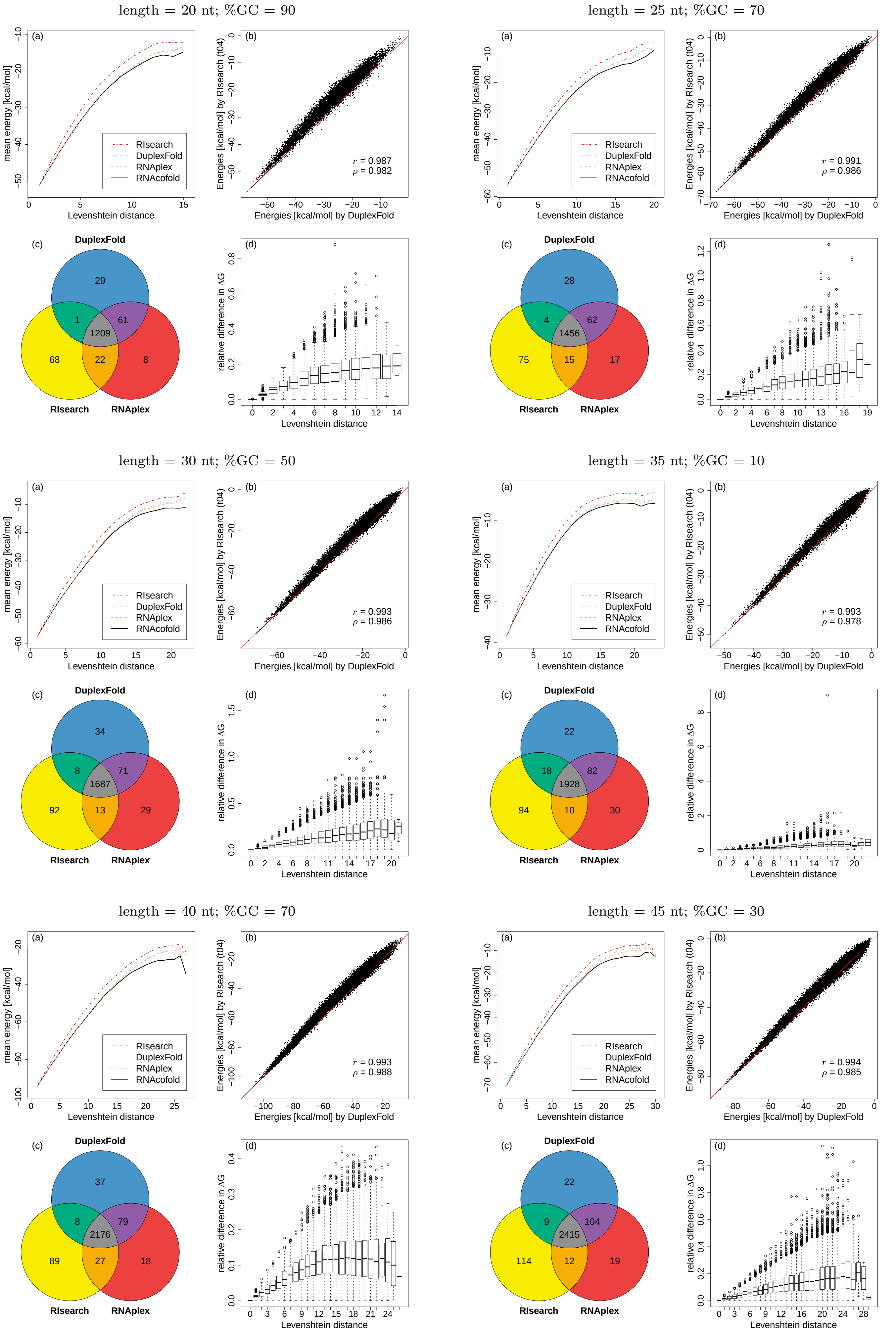


Figure 2: Caption on previous page.

# 2 Speed and memory benchmarks

## 2.1 RIsearch vs. RNAplex simple form

All measurements in this section were done with the GNU time command. Run times are given in seconds, the total amount of CPU time spend in user and kernel mode (%U + %S). For memory consumption we report the maximum resident set size (RSS) in Kilobytes (%M). There is a known bug in `time`, so this value is four times too large. However, the relative memory reduction is not affected. Values given in the following tables are what is reported by time without correction.

For `RNAplex` we specified parameter `-f 2`, so it uses the simple energy model in the backtracking as well, instead of the full energy model. For both tools, `RIsearch` and `RNAplex`, we set the per-nucleotide penalty to 0.3 kcal/mol. All other parameters were left at their default values.

| | RNAplex | | RIsearch | | comparison | |
|---|---|---|---|---|---|---|
| machine | time | mem | time | mem | time | mem |
| laptop | 24.55 | 5600 | 8.81 | 2288 | 2.79 | 2.45 |
| server | 12.64 | 5744 | 5.28 | 2448 | 2.39 | 2.35 |

Table 2: Comparison of runtime and memory requirements. Here we just repeated the 'Single Sequence Runtime benchmark' as given in the Supplementary Material to [7]. Query a set of 19 bacterial sRNAs (average length 131nt) in 100 target sequences of length 1200nt. time is runtime in seconds, mem is max RSS [kB], both as reported by `time` (U+S / M). For 'comparison' it is the speedup, respectively the memory reduction achieved by `RIsearch` in comparison to `RNAplex`. `RIsearch` was compiled with the default `-O3`; `RNAplex` was compiled with default parameters. Additionally, `RNAplex` was compiled specifying `-O3`, but performance did not improve. The laptop used has a Intel® Core™2 Duo CPU P8700 with 2.53 GHz, the server a Intel® Xeon® CPU X5570 with 2.93 GHz.

| #query | RNAplex | RIsearch | speedup |
|---|---|---|---|
| 1 | 17201.00 | 94.91 | 181.23 |
| 5 | 18547.07 | 472.18 | 39.28 |
| 100 | 39988.77 | 9093.04 | 4.40 |
| 500 | 130301.59 | 45598.25 | 2.86 |
| 1223 | 288182.65 | 112141.20 | 2.57 |

Table 3: Runtime on large genomic sequence. The complete human chromosome 1 without any filtering (249,250,621 nt) was used as target. The first column shows the number of miRNA sequences in the query set, up to 1223 which comprises all human mature miRNAs in miRBase 16. The second and third column give the runtime in seconds, the relative speedup of `RIsearch` is given in the last column. Memory consumption was reduced by a factor of 1.44 in all cases. From the data it seems that `RNAplex` uses much more time for the initialization. If cleaned for that, simply by subtracting the runtimes for a single microRNA, the speedup ranges between 2.4 and 3.6. Both tools compiled with `-O 3` optimization.

| seq lengths[nt] | | RNAplex | | RIsearch | | comparison | |
|---|---|---|---|---|---|---|---|
| query | target | time | mem | time | mem | time | mem |
| 10 | 1E+3 | 0.00 | 5520 | 0.00 | 2304 | NaN | 2.40 |
| 10 | 1E+4 | 0.00 | 5856 | 0.00 | 2624 | NaN | 2.23 |
| 10 | 1E+5 | 0.04 | 10544 | 0.01 | 6176 | 4.00 | 1.71 |
| 10 | 1E+6 | 0.68 | 56384 | 0.18 | 37952 | 3.78 | 1.49 |
| 10 | 1E+7 | 29.77 | 513408 | 1.89 | 354336 | 15.75 | 1.45 |
| 10 | 1E+8 | 2870.63 | 5083744 | 19.16 | 3518416 | 149.82 | 1.44 |
| 10 | 1E+9 | 321120.59 | 50786864 | 186.02 | 35159040 | 1726.27 | 1.44 |
| 25 | 1E+3 | 0.00 | 5536 | 0.00 | 2304 | NaN | 2.40 |
| 25 | 1E+4 | 0.01 | 5856 | 0.00 | 2640 | NaN | 2.22 |
| 25 | 1E+5 | 0.11 | 10528 | 0.04 | 6176 | 2.75 | 1.70 |
| 25 | 1E+6 | 1.36 | 56400 | 0.44 | 37936 | 3.09 | 1.49 |
| 25 | 1E+7 | 36.70 | 513408 | 4.50 | 354336 | 8.16 | 1.45 |
| 25 | 1E+8 | 2843.67 | 5083744 | 45.35 | 3518432 | 62.70 | 1.44 |
| 25 | 1E+9 | 274312.68 | 50786848 | 437.99 | 35159040 | 626.30 | 1.44 |
| 100 | 1E+3 | 0.00 | 5632 | 0.00 | 2384 | NaN | 2.36 |
| 100 | 1E+4 | 0.04 | 5904 | 0.01 | 2672 | 4.00 | 2.21 |
| 100 | 1E+5 | 0.41 | 10560 | 0.17 | 6160 | 2.41 | 1.71 |
| 100 | 1E+6 | 4.41 | 56368 | 1.73 | 37952 | 2.55 | 1.49 |
| 100 | 1E+7 | 67.41 | 513408 | 17.47 | 354352 | 3.86 | 1.45 |
| 100 | 1E+8 | 3158.54 | 5083760 | 176.02 | 3518416 | 17.94 | 1.44 |
| 100 | 1E+9 | 274929.19 | 50786864 | 1693.63 | 35159040 | 162.33 | 1.44 |
| 1000 | 1E+3 | 0.04 | 5840 | 0.01 | 2480 | 4.00 | 2.35 |
| 1000 | 1E+4 | 0.41 | 6144 | 0.17 | 2800 | 2.41 | 2.19 |
| 1000 | 1E+5 | 4.07 | 10544 | 1.70 | 6176 | 2.39 | 1.71 |
| 1000 | 1E+6 | 40.93 | 56400 | 17.06 | 37952 | 2.40 | 1.49 |
| 1000 | 1E+7 | 444.54 | 513392 | 170.71 | 354336 | 2.60 | 1.45 |
| 1000 | 1E+8 | 7330.30 | 5083744 | 1671.38 | 3518416 | 4.39 | 1.44 |
| 1000 | 1E+9 | 320057.08 | 50786848 | 16612.16 | 35159040 | 19.27 | 1.44 |

Table 4: Speed and memory benchmark on randomly generated sequences of different lengths. Columns 1 and 2 give the length (in nucleotides) of the query and target sequences respectively. Columns 3 and 4 list time (*user* + *system*) in seconds and memory requirements (maximum RSS [kB]), both as reported by the `time` command for `RNAplex`. Columns 5 and 6 show the same for `RIsearch`. Columns 7 and 8 show the improvement of `RIsearch` over `RNAplex`. `RIsearch` is at least 2.39 times as fast in all measurable cases. Here, `RNAplex` performed better with the default compiler flags again, *i.e.*, not specifying `-O 3`.

## 2.2 Using accessibility information

In order to run `RNAplex -a`, accessibility profiles need to be computed with `RNAplfold`. As memory requirements for the larger chromosomes exceeded our recources, we used human chromosome 21 here (the smallest one). With the recommended settings, `RNAplfold` runs more than 26 hours and uses more than 24 GiB to compute the accessibility profiles. The subsequent run of RNAplex in its current implementation also is more resource-demanding when making use of this information in comparison to the simple version. Screening this target with 5 miRNAs as query (as in Supplementary Table 3) takes:

| | time (minutes) | mem (GiB) |
|---|---|---|
| RNAplex -a (with accessibility) | 27.1 | 8.1 |
| RNAplex -c 30 (w/o accessibility) | 13.6 | 0.6 |
| RIsearch -d 30 | 1.7 | 0.4 |

# 3 Performance on known bacterial sRNA interactions

| | Sensitivity | | | | PPV | | | | F-measure | | | | MCC | | | |
|---|---|---|---|---|---|---|---|---|---|---|---|---|---|---|---|---|
| sRNA-mRNA | plex-a | plex-c | RIs99 | RIs04 | plex-a | plex-c | RIs99 | RIs04 | plex-a | plex-c | RIs99 | RIs04 | plex-a | plex-c | RIs99 | RIs04 |
| GcvB-gltI* | 0.923 | 0.846 | 1.000 | *1.000* | 1.000 | 0.393 | 0.448 | *0.448* | 0.960 | 0.537 | 0.619 | *0.619* | 0.961 | 0.577 | 0.670 | *0.670* |
| GcvB-argT* | 0.875 | *1.000* | *1.000* | 1.000 | 0.824 | *0.800* | *1.000* | 1.000 | 0.848 | *0.889* | *1.000* | 1.000 | 0.849 | *0.894* | *1.000* | 1.000 |
| GcvB-dppA | 1.000 | 0.941 | 0.941 | 0.941 | 0.515 | 0.485 | 0.500 | 0.485 | 0.680 | 0.640 | 0.653 | 0.640 | 0.718 | 0.676 | 0.686 | 0.676 |
| GcvB-livJ | 0.955 | 1.000 | 1.000 | 1.000 | 0.955 | 1.000 | 1.000 | 1.000 | 0.955 | 1.000 | 1.000 | 1.000 | 0.955 | 1.000 | 1.000 | 1.000 |
| GcvB-livK* | 1.000 | *1.000* | 1.000 | *1.000* | 0.565 | *0.565* | 0.481 | *0.929* | 0.722 | *0.722* | 0.650 | *0.963* | 0.752 | *0.752* | 0.694 | *0.964* |
| GcvB-oppA | 1.000 | 1.000 | 1.000 | 1.000 | 0.957 | 0.957 | 1.000 | 1.000 | 0.978 | 0.978 | 1.000 | 1.000 | 0.978 | 0.978 | 1.000 | 1.000 |
| GcvB-STM4351 | 0.889 | *1.000* | *1.000* | *1.000* | 0.348 | *0.409* | *1.000* | *1.000* | 0.500 | *0.581* | *1.000* | *1.000* | 0.556 | *0.640* | *1.000* | *1.000* |
| MicA-lamB | *1.000* | *1.000* | 1.000 | 1.000 | *0.821* | *1.000* | 1.000 | 1.000 | *0.902* | *1.000* | 1.000 | 1.000 | *0.906* | *1.000* | 1.000 | 1.000 |
| MicA-ompA | 1.000 | 1.000 | 0.938 | 1.000 | 1.000 | 1.000 | 1.000 | 1.000 | 1.000 | 1.000 | 0.968 | 1.000 | 1.000 | 1.000 | 0.968 | 1.000 |
| DsrA-rpoS* | 0.571 | 1.000 | 1.000 | 1.000 | 0.667 | 0.778 | 0.778 | 0.778 | 0.615 | 0.875 | 0.875 | 0.875 | 0.617 | 0.882 | 0.882 | 0.882 |
| RprA-rpoS | 0.316 | 0.579 | 0.579 | 0.579 | 0.286 | 1.000 | 1.000 | 1.000 | 0.300 | 0.733 | 0.733 | 0.733 | 0.300 | 0.761 | 0.761 | 0.761 |
| IstR-tisA* | 1.000 | 1.000 | 1.000 | 1.000 | 1.000 | 0.821 | 0.821 | 0.821 | 1.000 | 0.902 | 0.902 | 0.902 | 1.000 | 0.906 | 0.906 | 0.906 |
| MicC-ompC | 0.727 | *0.727* | *0.727* | *0.727* | 1.000 | *1.000* | *1.000* | *1.000* | 0.842 | *0.842* | *0.842* | *0.842* | 0.853 | *0.853* | *0.853* | *0.853* |
| MicF-ompF | 0.800 | 0.960 | *0.800* | 0.960 | 0.952 | 0.960 | *0.952* | 0.960 | 0.870 | 0.960 | *0.870* | 0.960 | 0.873 | 0.960 | *0.873* | 0.960 |
| RyhB-sdhD | 0.588 | 0.794 | 0.676 | 0.676 | 1.000 | 0.964 | 0.852 | 0.852 | 0.741 | 0.871 | 0.754 | 0.754 | 0.767 | 0.875 | 0.759 | 0.759 |
| RyhB-sodB | 1.000 | 1.000 | 1.000 | 1.000 | 0.450 | 0.818 | 1.000 | 1.000 | 0.621 | 0.900 | 1.000 | 1.000 | 0.671 | 0.905 | 1.000 | 1.000 |
| SgrS-ptsG | 0.739 | 0.739 | *0.739* | *0.739* | 1.000 | 1.000 | *1.000* | *1.000* | 0.850 | 0.850 | *0.850* | *0.850* | 0.860 | 0.860 | *0.860* | *0.860* |
| SF, −subopt | 0.787 | 0.639 | 0.655 | 0.656 | 0.736 | 0.599 | 0.581 | 0.641 | 0.734 | 0.603 | 0.597 | 0.639 | 0.748 | 0.611 | 0.607 | 0.644 |
| SF, +subopt | 0.846 | 0.917 | 0.906 | 0.919 | 0.785 | 0.821 | 0.873 | 0.898 | 0.787 | 0.840 | 0.866 | 0.891 | 0.801 | 0.854 | 0.877 | 0.899 |
| LF, −subopt | 0.703 | 0.592 | 0.604 | 0.620 | 0.779 | 0.609 | 0.614 | 0.651 | 0.715 | 0.590 | 0.599 | 0.626 | 0.728 | 0.595 | 0.604 | 0.631 |
| LF, +subopt | 0.762 | 0.836 | 0.805 | 0.818 | 0.827 | 0.878 | 0.905 | 0.931 | 0.768 | 0.844 | 0.834 | 0.850 | 0.781 | 0.850 | 0.844 | 0.862 |

Table 5: Prediction accuracy. Sensitivity (also recall or true positive rate (TPR)), positive predictive value (PPV) or precision, F-measure, and MCC (harmonic and geometric mean of the first two) were calculated for the set of 17 experimentally verified sRNA-mRNA interactions. Averages are given in the last four lines. We tested `RNAplex` using precomputed accessibility profiles (plex-a), as well as the basic version (plex-c) with per-nucleotide penalty `-c 30`. RIs99 and RIs04 stand for `RIsearch` using 1999 and 2004 parameter set, respectively, also with per-nucleotide penalty of 30 (= 0.3 kcal/mol). Numbers shown in gray italics refer to interactions that have not been found as the single best-scoring, but only when taking into account suboptimal solutions. For interactions marked with an asterisk (*), we have extracted a longer and a shorter version from the literature. For example, [8] identified residues that were protected in *in vitro* footprinting experiments and extended the target sites by biocomputational predictions. For this table we used the shorter forms (SF, with boundaries as given in Main Table 1). When instead using the longer forms (LF, maximum numbers of pairs shown in the original papers), we get the average measures as reported in the last two lines. When excluding suboptimal solutions (−subopt), `RNAplex` with accessibility misses only one interaction, while the other methods miss five each. When the top prediction does not share a base pair with the experimentally verified location, they contribute with 0 to the average. In all these cases it is enough to look at the three best suboptimal solutions in order to find one that overlaps the verified location. When allowing these suboptimal solutions (+subopt), the values as printed in gray italics contribute to the average.

# 4 Identifying human miRNA targets on chromosome-scale

## 4.1 Ranking known targets

| interaction pair | | chromosome | | RIsearch | | RNAplex | | GUUGle | | GUUGle* | | TargetScanS | | miRanda | | GUUGle* ∩ | |
|---|---|---|---|---|---|---|---|---|---|---|---|---|---|---|---|---|---|
| mRNA | miRNA | name | non-N nt | thr | count | thr | count | thr | count | thr | count | thr | count | thr | count | RIsearch | RNAplex |
| AGTR1 | miR-155 | 3 + | 97,261,371 | -13.25 | 113,671 | -14.37 | 180,386 | 8 | 570,135 | 8 | 39,957 | 0.073 | 9,800 | -13.52 | 8,492 | 11,561 | 15,607 |
| BCL2 | miR-16 | 18 – | 39,669,911 | -18.48 | 718 | -18.90 | 3,843 | 10 | 15,346 | 8 | 9,291 | -0.023 | 1,969 | -21.84 | 238 | 364 | 1,305 |
| SLC7A1 | miR-122 | 13 – | 49,987,579 | -22.88 | 125 | -23.80 | 290 | 9 | 233,723 | 8 | 125,504 | -0.031 | 1,098 | -25.44 | 110 | 74 | 145 |
| TPPP3 | miR-16 | 16 – | 39,076,387 | -19.41 | 584 | -20.80 | 1,505 | 7 | 295,580 | 7 | 33,455 | -0.015 | 1,641 | -19.22 | 1,209 | 310 | 602 |
| CLOCK | miR-141 | 4 – | 92,263,291 | -14.93 | 21,192 | -16.40 | 33,745 | 9 | 189,863 | 7 | 37,234 | 0.036 | 5,302 | -19.74 | 1,468 | 2,637 | 3,251 |
| CXCL12 | miR-23a | 10 – | 67,660,041 | -18.01 | 12,188 | -19.80 | 12,591 | 8 | 345,446 | 8 | 10,948 | 0.028 | 866 | -15.10 | 4,559 | 2,153 | 2,341 |
| CYP1B1 | miR-27b | 2 – | 123,169,357 | -26.94 | 27 | -28.20 | 32 | 12 | 8,253 | 7 | 130,018 | 0.012 | 4,742 | -30.62 | 13 | 19 | 25 |
| E2F3 | miR-34a | 6 + | 85,195,913 | -18.53 | 45,062 | -17.20 | 226,049 | 9 | 219,103 | 8 | 79,646 | -0.162 | 65 | -17.06 | 9,835 | 12,361 | 48,237 |
| EZH2 | miR-101 | 7 – | 77,676,101 | -15.45 | 3,659 | -16.90 | 7,342 | 9 | 69,204 | 8 | 9,164 | -0.051 | 388 | -19.29 | 712 | 1,031 | 1,510 |
| PARP8 | miR-145 | 5 + | 88,834,294 | -20.01 | 28,064 | -21.80 | 32,349 | 10 | 52,652 | 8 | 36,220 | NF | NF | -23.53 | 1,802 | 8,421 | 9,668 |
| FSTL1 | miR-206 | 3 – | 97,261,371 | -15.08 | 67,128 | -18.40 | 31,741 | 8 | 1,221,194 | 7 | 528,477 | 0.054 | 9,023 | -13.85 | 14,297 | 19,652 | 10,410 |
| GJA1 | miR-1 | 6 + | 85,195,913 | -11.65 | 36,595 | -14.30 | 23,215 | 8 | 725,891 | 8 | 136,280 | -0.017 | 1,309 | -15.70 | 3,443 | 15,783 | 10,648 |
| GJA1 | miR-206 | 6 + | 85,195,913 | -12.49 | 237,213 | -15.03 | 180,291 | 8 | 1,087,518 | 8 | 136,280 | -0.017 | 1,252 | -17.46 | 5,928 | 57,790 | 47,456 |
| HAND2 | miR-1 | 4 – | 92,263,291 | -12.14 | 29,151 | -12.20 | 106,885 | 8 | 868,628 | 8 | 162,332 | 0.004 | 2,971 | NF | NF | 13,501 | 42,709 |
| HOXA1 | miR-10a | 7 – | 77,676,101 | -12.29 | 162,987 | -15.93 | 75,673 | 8 | 285,837 | 8 | 13,477 | -0.046 | 1,932 | -15.93 | 5,329 | 21,756 | 12,681 |
| KIT | miR-221 | 4 + | 92,263,291 | -14.95 | 185,809 | -17.70 | 123,549 | 8 | 666,674 | 7 | 58,517 | -0.067 | 623 | -12.15 | 10,761 | 18,267 | 13,616 |
| KIT | miR-222 | 4 + | 92,263,291 | -15.20 | 134,277 | -15.25 | 301,413 | 7 | 667,971 | 7 | 58,517 | -0.088 | 291 | -10.33 | 7,020 | 14,707 | 24,752 |
| KRAS | let-7a | 12 – | 63,199,786 | -14.71 | 46,793 | -16.30 | 59,593 | 7 | 1,815,721 | 7 | 385,350 | NF | NF | NF | NF | 16,286 | 20,507 |
| LIN28A | let-7b | 1 + | 111,179,527 | -25.61 | 444 | -27.00 | 670 | 14 | 8,845 | 8 | 183,041 | -0.091 | 470 | -27.61 | 404 | 238 | 330 |
| MAPK14 | miR-24 | 6 + | 85,195,913 | -27.07 | 154 | -27.10 | 653 | 8 | 290,052 | 8 | 40,436 | -0.049 | 1,560 | -29.28 | 118 | 99 | 388 |
| MYCN | miR-101 | 2 + | 123,169,357 | -12.11 | 58,119 | -13.85 | 90,266 | 9 | 111,230 | 8 | 14,442 | -0.062 | 409 | -16.11 | 3,785 | 9,659 | 11,584 |
| NRAS | let-7a | 1 – | 111,179,527 | -13.20 | 216,020 | -17.70 | 50,049 | 9 | 525,120 | 8 | 181,621 | -0.046 | 1,805 | -14.33 | 17,668 | 61,405 | 19,476 |
| PTEN | miR-19a | 10 + | 67,660,041 | -16.38 | 1,769 | -17.70 | 3,779 | 10 | 11,427 | 8 | 20,980 | -0.094 | 11 | -17.97 | 700 | 751 | 1,438 |
| ARHGAP32 | miR-132 | 11 – | 64,323,812 | -18.11 | 11,583 | -18.80 | 38,484 | 10 | 11,319 | 7 | 44,392 | 0.022 | 1,844 | -18.43 | 1,960 | 1,157 | 2,569 |
| SMC1A | let-7e | X – | 59,371,681 | -21.33 | 1,629 | -22.20 | 3,416 | 12 | 33,335 | 8 | 106,473 | -0.009 | 2,677 | -24.08 | 812 | 812 | 1,577 |
| TMSB4X | miR-1 | X + | 59,371,681 | -16.00 | 925 | -16.90 | 2,168 | 9 | 171,143 | 8 | 92,463 | 0.048 | 5,123 | -18.72 | 444 | 504 | 1,157 |
| TPM1 | miR-21 | 15 + | 41,621,622 | -13.28 | 30,154 | -15.60 | 29,478 | 9 | 50,347 | 8 | 17,875 | 0.016 | 1,206 | NF | NF | 4,913 | 4,613 |
| Rank product | | | | | 4.59 | | 5.66 | | 7.48 | | 6.17 | | **2.15** | | 2.17 | 2.23 | 3.24 |
| Relative hit score | | | | | 22.48 | | 20.62 | | 3.81 | | 16.40 | | 24.61 | | 23.63 | **26.09** | 25.83 |

Table 6: The table shows the interacting gene and miRNA, with chromosome information for target site location (name, strand, and the number of bases that have not been masked). For each tool, we report a threshold (thr) found by looking for the highest scoring hit that overlaps a verified interaction site of this mRNA-miRNA pair. For `RIsearch` and `RNAplex` this threshold is the $\Delta G$, for `GUUGle` the match length, for `TargetScanS` the context+ score, for `miRanda` again the energy. As count we report the number of hits (of the given miRNA within this chromosome, direction) that fulfill this threshold, *i.e.*, all predictions that score at least as good as the best verified interaction for that pair. `GUUGle*` uses only the seed of the miRNA as query (nt 1–8) instead of the whole mature miRNA (in `GUUGle`). For `RIsearch` and `RNAplex` we additionally intersected the hits with those from `GUUGle*` and present the counts in the last two columns (number of predictions that overlap complete `GUUGle` seed matches and fulfill the energy threshold as applied for `RIsearch` and `RNAplex` respectively). NF stands for 'not found'. We use two different methods to evaluate performance and give the results in the last two rows where the best result is highlighted in bold.

## 4.2 Efficacy of RIsearch as filter

| interaction pair | | TargetScanS | | | | miRanda | | | |
|---|---|---|---|---|---|---|---|---|---|
| mRNA | miRNA | unfiltered | G* | RIs | G*∩RIs | unfiltered | G* | RIs | G*∩RIs |
| AGTR1 | miR-155 | 13,575 | 0.00% | 57.51% | 57.67% | 16,753 | 18.24% | 49.41% | 58.82% |
| BCL2 | miR-16 | 6,212 | 0.00% | 16.85% | 17.06% | 7,147 | 9.49% | 20.99% | 27.12% |
| SLC7A1 | miR-122 | 4,752 | 0.00% | 5.35% | 5.89% | 6,139 | 9.09% | 7.31% | 17.66% |
| TPPP3 | miR-16 | 7,384 | 0.00% | 15.28% | 15.59% | 7,599 | 8.19% | 17.70% | 23.41% |
| CLOCK | miR-141 | 15,922 | 0.00% | 47.46% | 47.61% | 14,890 | 18.66% | 38.72% | 50.64% |
| CXCL12 | miR-23a | 14,275 | 28.13% | 56.39% | 64.57% | 11,865 | 8.42% | 41.93% | 47.91% |
| CYP1B1 | miR-27b | 23,204 | 0.00% | 23.59% | 23.83% | 19,190 | 10.78% | 16.08% | 24.17% |
| E2F3 | miR-34a | 10,783 | 0.00% | 1.28% | 2.14% | 12,938 | 9.90% | 4.04% | 15.82% |
| EZH2 | miR-101 | 8,447 | 0.00% | 68.06% | 68.08% | 6,394 | 14.65% | 33.47% | 44.21% |
| PARP8 | miR-145 | NF | NF | NF | NF | 15,476 | 11.45% | 25.85% | 35.67% |
| FSTL1 | miR-206 | 15,069 | 0.00% | 25.78% | 25.99% | 19,702 | 9.00% | 30.56% | 38.80% |
| GJA1 | miR-1 | 12,865 | 0.00% | 67.40% | 67.44% | 18,334 | 9.64% | 60.36% | 65.04% |
| GJA1 | miR-206 | 12,865 | 0.00% | 26.37% | 26.55% | 17,304 | 9.32% | 30.80% | 38.86% |
| HAND2 | miR-1 | 13,614 | 0.00% | 65.60% | 65.65% | NF | NF | NF | NF |
| HOXA1 | miR-10a | 7,548 | 0.00% | 10.57% | 10.73% | 7,507 | 17.72% | 14.11% | 27.24% |
| KIT | miR-221 | 9,406 | 35.73% | 22.02% | 44.78% | 11,952 | 21.81% | 16.60% | 31.41% |
| KIT | miR-222 | 9,406 | 35.73% | 29.13% | 51.57% | 7,777 | 10.17% | 28.79% | 35.64% |
| KRAS | let-7a | NF | NF | NF | NF | NF | NF | NF | NF |
| LIN28A | let-7b | 11,539 | 0.00% | 7.04% | 8.28% | 20,788 | 10.03% | 9.04% | 18.30% |
| MAPK14 | miR-24 | 10,229 | 0.00% | 2.38% | 2.39% | 11,080 | 8.03% | 7.58% | 16.25% |
| MYCN | miR-101 | 13,408 | 0.00% | 67.62% | 67.70% | 10,340 | 14.72% | 34.61% | 45.34% |
| NRAS | let-7a | 11,446 | 0.00% | 13.68% | 13.91% | 20,290 | 9.23% | 13.03% | 21.45% |
| PTEN | miR-19a | 8,299 | 0.00% | 54.40% | 54.58% | 10,680 | 19.58% | 56.77% | 65.38% |
| ARHGAP32 | miR-132 | 7,378 | 0.00% | 50.76% | 50.95% | 6,166 | 17.74% | 26.45% | 40.33% |
| SMC1A | let-7e | 6,288 | 0.00% | 9.27% | 9.83% | 11,564 | 10.78% | 12.92% | 23.74% |
| TMSB4X | miR-1 | 9,227 | 0.00% | 67.42% | 67.49% | 12,742 | 10.38% | 60.57% | 65.24% |
| TPM1 | miR-21 | 3,968 | 0.00% | 70.14% | 70.21% | NF | NF | NF | NF |
| Average | | 10,684 | 3.98% | 35.25% | 37.62% | 12,692 | 12.38% | 27.40% | 36.60% |
| G full | | | 3.98% | | 36.93% | | 2.56% | | 29.83% |

Table 7: `RIsearch` as pre-filter. For each of the interactions, we report the *unfiltered* number of predictions made by `TargetScanS` and `miRanda`, together with the relative reduction achieved by different (combination of) tools. G*: `GUUGle*` as described in previous table; RIs: `RIsearch` with a threshold of -11 kcal/mol; G*∩RIs: combination of both. Interactions that are denoted as not found (*NF*), have been found by the filter, but not by the respective method. Results are summarized as their averages below. The last row shows the according averages, when using `GUUGle` instead of `GUUGle*`.

These results show that `GUUGle` can hardly reduce `TargetScanS` candidates. This is because `TargetScanS` uses an even stricter seed requirement, all candidates identified by `TargetScanS` are also found by `GUUGle`. The only exception is the "7mer-1a" criterion, an exact match to positions 2–7 of the mature miRNA (the seed) followed by an '`A`'. In these cases, a perfect complementary stretch of six nucleotides is sufficient to be considered as candidate for `TargetScanS`. There are only three interactions where `GUUGle` in fact reduces the number of candidates.

For `miRanda` the trend is not as strong, but also here we see that `RIsearch` alone achieves a bigger candidate reduction than `GUUGle` alone. As both tools filter out different candidates, their combined effect is strongest.

The reductions that can be achieved by `RIsearch` as a pre-filter differ widely (see Suppl. Table 7, e.g. for `TargetScanS` relative reduction varies between 1% and 70%).
Part of the explanation is the difference in GC-content of the mature miRNA sequences. The higher the GC-content, the more likely are low binding energies. With the conservative threshold of -11 kcal/mol, the list of candidates can not be reduced substantially in those cases. One could address this, by choosing a stricter cut-off for miRNAs with a potentially stronger interaction.
This relation can be seen in the figure to the right. The Pearson correlation coefficient $r$ between the GC-content and the reduction in `miRanda` hits is -0.6595 (p-value: 0.00046) and for `TargetScanS` -0.6996 (p-value: 9.953e-5).

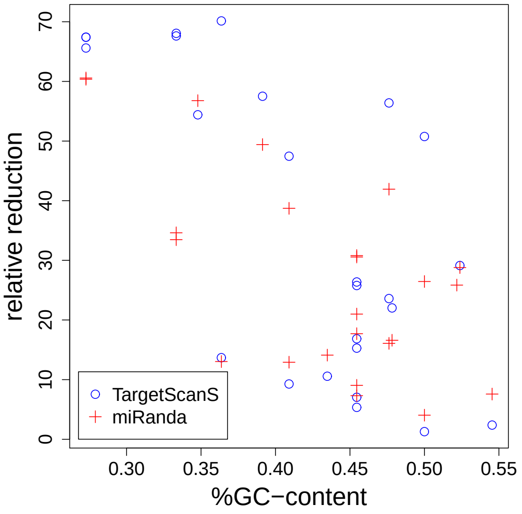


Figure 3: Effect of GC-content of the miRNA sequence on relative reduction achieved.

# References


[1] D. H. Mathews, M. D. Disney, J. L. Childs, S. J. Schroeder, M. Zuker, and D. H. Turner, "Incorporating chemical modification constraints into a dynamic programming algorithm for prediction of RNA secondary structure.," *Proc Natl Acad Sci U S A*, vol. 101, pp. 7287–7292, May 2004.

[2] M. Rehmsmeier, P. Steffen, M. Höchsmann, and R. Giegerich, "Fast and effective prediction of microRNA/target duplexes," *RNA*, vol. 10, no. 10, pp. 1507–17, 2004.

[3] J. S. Reuter and D. H. Mathews, "RNAstructure: software for RNA secondary structure prediction and analysis.," *BMC Bioinformatics*, vol. 11, p. 129, 2010.

[4] S. H. Bernhart, H. Tafer, U. Mückstein, C. Flamm, P. F. Stadler, and I. L. Hofacker, "Partition function and base pairing probabilities of RNA heterodimers.," *Algorithms Mol Biol*, vol. 1, no. 1, p. 3, 2006.

[5] N. O. Hodas and D. P. Aalberts, "Efficient computation of optimal oligo-RNA binding.," *Nucleic Acids Res*, vol. 32, no. 22, pp. 6636–6642, 2004.

[6] M. Andronescu, R. Aguirre-Hernández, A. Condon, and H. H. Hoos, "RNAsoft: A suite of RNA secondary structure prediction and design software tools.," *Nucleic Acids Res*, vol. 31, pp. 3416–3422, Jul 2003.

[7] H. Tafer, F. Amman, F. Eggenhofer, P. F. Stadler, and I. L. Hofacker, "Fast accessibility-based prediction of RNA–RNA interactions," *Bioinformatics*, vol. 27, no. 14, pp. 1934–1940, 2011.

[8] C. M. Sharma, F. Darfeuille, T. H. Plantinga, and J. Vogel, "A small RNA regulates multiple ABC transporter mRNAs by targeting C/A-rich elements inside and upstream of ribosome-binding sites.," *Genes Dev*, vol. 21, pp. 2804–2817, Nov 2007.
